# Male‐Like Plumage in an Urban Nesting Veraguan Mango: Evidence of a Female‐Limited Polymorphism?

**DOI:** 10.1002/ece3.72071

**Published:** 2025-08-31

**Authors:** Dallas R. Levey, Gretchen C. Daily

**Affiliations:** ^1^ Department of Biology Stanford University Stanford California USA

**Keywords:** Costa Rica, female‐limited polymorphism, male mimicry, nonsexual social selection, Trochilidae

## Abstract

A first instance of parental care by a male‐plumaged hummingbird from a sexually dimorphic species contributes important natural history understanding and helps illuminate the possibility of interesting female‐limited polymorphisms across hummingbird species. Using photos and 2.5 min of video taken at close proximity, we documented a Veraguan mango ( *Anthracothorax veraguensis* ) with male plumage both incubating eggs and later feeding nestlings in the town of Palmar Norte in southern Costa Rica. Based on plumage characteristics and range, we rule out the similar green‐breasted mango ( *A. prevostii* ) that occurs in close geographic proximity. On‐ground exploration and Google Earth imagery revealed the landscape surrounding the nest as a heterogeneous mix of urban, residential, and agricultural land. Given our assumptions about the bird's sex and age, we speculated on potential mechanisms for male‐like plumage in adult female Veraguan mango (and related species in the *Anthracothorax* genus), including age‐related plumage ontogeny and the interaction of social and ecological selection pressures. Our observation contributes valuable information to the natural history of the Veraguan mango and opens the possibility of a female‐limited polymorphism in the species.

## Graphical abstract

We document the first known case of a male‐plumaged Veraguan mango ( *Anthracothorax veraguensis* ) providing parental care, supported by photographs and video from southern Costa Rica. The bird incubated eggs and fed nestlings in a nest located within a heterogeneous urban–agricultural landscape. We discuss possible mechanisms for a potential female‐limited plumage polymorphism, considering prior observations in related hummingbird species and the ecological and social pressures that may influence its occurrence.

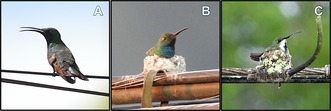

## 1 Introduction

In sexually dimorphic hummingbird species, it is a curiosity to find adult females with adult male plumage. Previous research assessing museum specimens has suggested that ~25% of ~300 hummingbird species show evidence of adult females with male plumage (Bleiweiss 1992, 2001; Diamant et al. 2021). However, the difficult task of discerning females with male plumage from skins has called into question the frequency of the phenomenon across hummingbirds (Clark 2022; Clark et al. 2022). Evidence from live birds suggests that ~1% of hummingbird species show repeated incidents of male‐plumaged birds from sexually dimorphic species providing parental care. These species include the white‐necked jacobin ( *Florisuga mellivora* ; Elgar 1978; Schuchmann 1999; Falk et al. 2021; Falk, Castaño‐Diaz, et al. 2025), green‐breasted mango ( *Anthracothorax prevostii* ; Stiles et al. 1989), and black‐throated mango ( *A. nigricollis* ; Quesnel 1995; Rochford 2012; Cueva and Celorio Mosquera 2025).

We present photo and video evidence of a male‐plumaged Veraguan mango (*Antracothorax veraguensis*) providing parental care in southern Costa Rica. We describe plumage characteristics of the hummingbird, the behaviors recorded in the video, and the landscape context. While acknowledging the limitations of a single observation, we consider potential mechanisms to explain the event.

## 2 Material and Methods

### 2.1 Study Area

We observed a putative adult female with adult male plumage in Palmar Norte, a small town in the Puntarenas province of Costa Rica (8°57′55.8″ N, 83°27′42.5″ W; 32 MSL). On a landscape scale, Palmar Norte is located north of Río Térraba and at the southern base of a coastal mountain range. Dominant land cover types in the area include oil palm plantations, cattle pasture, lowland tropical rainforest patches, residential gardens, and urbanized towns. We found the nest approximately 5.5 m above ground level, adhered with cobweb or plant fibers to a powerline cable running alongside the northern edge of Carretera Costanera Sur and over the exit of a supermarket parking lot. During the observation, semi‐truck, car, and motorcycle traffic on Carretera Costanera Sur was constant and loud. The habitat context immediately about the nest location included a mix of paved surfaces, grassy lots, residential gardens, cattle pasture, and live fences of native and nonnative trees.

### 2.2 Determining Species Identification, Age, and Sex

**FIGURE 1:**
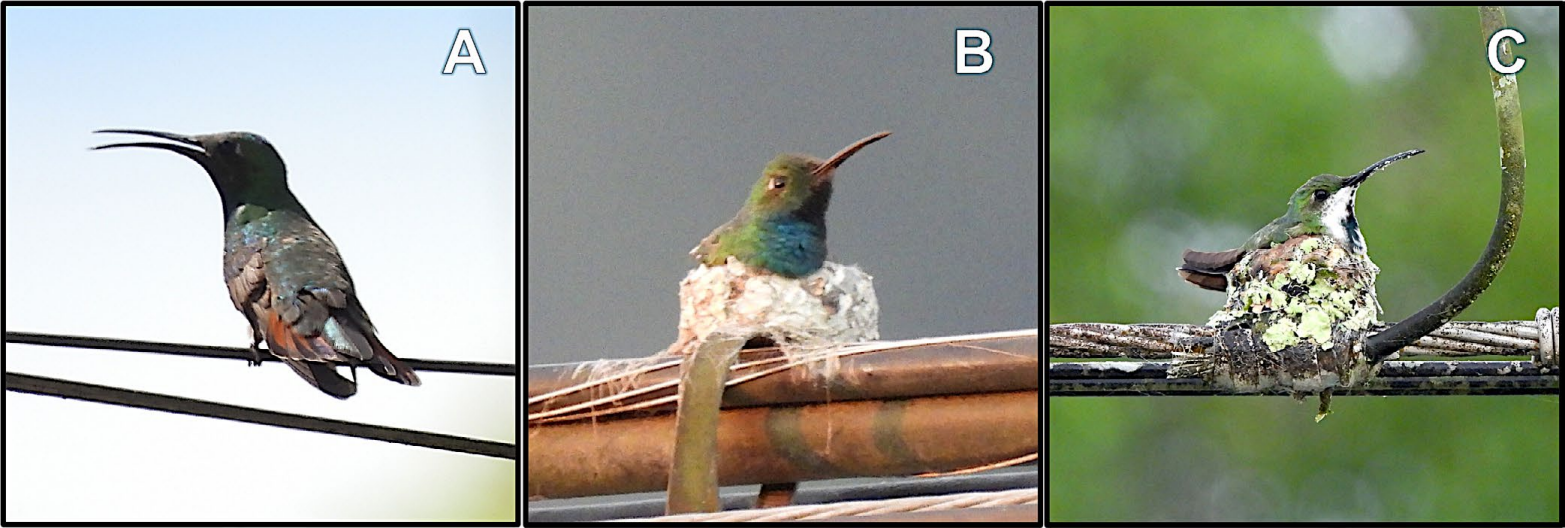
Photo series of the putative adult female Veraguan mango ( *Anthracothorax veraguensis* ) with male‐like plumage providing parental care (A, B) and an example of a typical adult female Veraguan mango on a nest (C). In photos A and B (taken on February 5, 2025), the putative female is perched on a cable and nest, showing two angles of the plumage. The hummingbird's overall size and proportions are typical of mangoes, including a long tail, stout, black bill with a downward curvature, and primarily green colored. The tail is mostly maroon with black terminal banding and thin white bordering, the latter typical of adult females. The throat is blue and darkest in a wedge‐shaped area that is restricted to the throat. In photo C (taken on September 30, 2024, near Ciudad Neily, Costa Rica), we provide a photo of an adult Veraguan mango with typical adult female plumage to demonstrate the plumage differences, including the contrasting white undersides with a dark vertical stripe down the throat. Photos by DRL.

**FIGURE 2:**
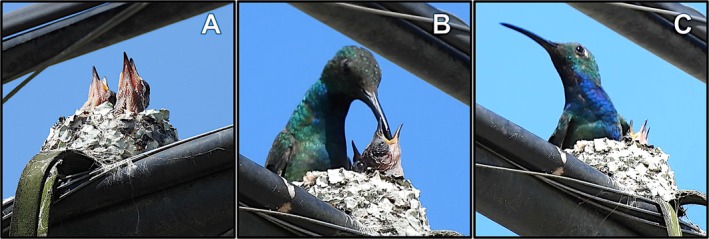
Photo series of the putative adult female Veraguan mango ( *Anthracothorax veraguensis* ) with male‐like plumage (in Figure 1A,B) feeding young in the outskirts of Palmar Norte, Costa Rica. Photos A–C (taken February 13, 2025) show the nestlings in begging posture and being fed. Photo C shows the putative adult female on the rim of the nest with the two nestlings and provides a close‐up view of the underside plumage, including the blue throat color. Photos by DRL.

For the species identification, we used range and the characteristics of the male‐like plumage. Phylogenetically, the Veraguan mango is in a clade with the Hispaniolan mango ( *A. dominicus* ), green‐breasted mango, and black‐throated mango ( *A. nigricollis* ; Schmitz‐Ornés and Haase 2009; Schuchmann and Boesman 2020). Two species of mango within this clade occur in Costa Rica: the green‐breasted mango, which is distributed in the lowlands on both coasts and in the highlands of the Central Valley, and the Veraguan mango, whose northernmost distribution begins near the southern limit of the green‐breasted mango and extends along the Pacific lowlands throughout the Osa Peninsula in Costa Rica and into Panama (Ridgely and Gwynne 1989; Garrigues and Dean 2007; Schuchmann and Boesman 2020). The observed nesting behavior on a powerline in urban habitat with nearby agricultural land, residential gardens, and some native forest is noted occasionally for the Veraguan mango and other members of *Antracothorax*, though they typically nest conspicuously high above ground in tall trees at the forest edge or isolated in grassy habitats (Garrigues and Dean 2007; Schuchmann and Boesman 2020; Juárez et al. 2024; Cueva and Celorio Mosquera 2025). The key plumage trait separating the Veraguan mango from the similar green‐breasted mango is the lack of a vertical dark stripe in the center of the blue iridescent color that extends from the throat to the breast (Schuchmann 1999; Stiles et al. 1989; Schuchmann and Boesman 2020; Juárez et al. 2024). Some Veraguan mango males show a small dark green wedge on the throat that rarely extends to the breast like it does in the green‐breasted mango (Wetmore 1968; Olson 1993; Fagan and Komar 2016). The hummingbird showed a dark throat color in certain angles that did not appear to extend past the throat to the breast and belly at any angle (Figures 1 and 2).

As for age determination, the bird is likely an adult since the plumage lacks the contrasting white belly with a dark central vertical stripe that is typical of immature mangoes (Stiles et al. 1989; Figures 1 and 2). As for sex determination, we consider the bird a putative female using one morphological aspect and the parental care behavior while acknowledging that the latter has been observed rarely in male hummingbirds (Moore 1947). Morphologically, the bird has white tips on the outer retrices, which is an expected feature in adult females and immatures but not adult males (Figure 1; Schuchmann and Boesman 2020).

## 3 Results

### 3.1 Event Description

We observed, photographed, and recorded videos (https://doi.org/10.5061/dryad.jh9w0vtp7; Levey and Daily 2025) of a putative female Veraguan mango with male plumage incubating eggs and feeding nestlings in February 2025 (Figure 1A). Our first observation on February 5 at 4:56 p.m. included 45 min of viewing the unattended nest with binoculars and a Nikon COOLPIX P950 camera from 10 m away and 5 min of viewing the adult mango perched on the rim of the nest and incubating the eggs (Figure 1B,C). We did not see the adult tending to the nest structure or adding more materials (Video 1).

**VIDEO 1:** Video of the putative adult female Veraguan Mango ( *Anthracothorax veraguensis* ) with male plumage from Palmar Norte, Costa Rica feeding two nestlings (taken on February 13, 2025). We observed the nest from 10 m away using a Nikon COOLPIX P950 camera. The nest was adhered to a powerline with cobweb or plant fibers 5.5 m above ground alongside a major highway. The overall landscape included a heterogenous mix of urban habitats, cattle pasture, African oil palm plantations, residential gardens, live fences, and patches of remnant native forest. The putative female's plumage differs from typical adult female plumage in the Veraguan mango, which includes a dark green uppersides contrasting with white undersides and a dark, central vertical stripe running down the belly from the throat. Aside from the white tips to the outer retrices, which is an adult female trait, the plumage matches that of a typical adult male Veraguan mango, including bright blue coloration on the throat, chest, and upper breast. Video content can be viewed at https://onlinelibrary.wiley.com/doi/10.1002/ece3.72071.

We returned to view the nest on February 13 at 1:26 p.m. We observed the nest from 10 m away using binoculars and a Nikon COOLPIX P950 camera. We observed two nestlings in a food begging posture with open mouths pointed upwards (Figure 2A). We observed the unattended nestlings for 28 min. After this period, the putative female returned and perched on a nearby wire (Figure 1A). After 3 min of resting and preening, the putative female flew to the nest and perched on the rim to begin feeding the nestlings (Figure 2B). In total, the putative female spent 1 min and 50 s in a feeding bout that entailed eight pauses of 4.1 ± 1 s each (30% of total time) and nine feedings of 7.9 ± 2.4 s each (70% of total feeding bout time). The nestling on the left side of the putative female in the video received four full feedings for a total of 35 s (32% of total time), averaging 8.8 ± 2.2 s. The nestling on the right side of the putative female received five full and one partial feedings for a total of 44 s (40% of total time), averaging 7.3 ± 2.6 s. For 39 s after the last feeding, the putative female perched on the edge of the nest, looking left and right while occasionally reaching around the nest with her bill to grab at a lichen attached to the nest wall or to reach at one of the nestlings (Figure 2C). Afterward, the putative female flew away from the nest.

## 4 Discussion

Our observation of a male‐plumaged Veraguan mango incubating and feeding nestlings constitutes evidence for the third species in the *Anthracothorax* genus to have cases of male‐plumaged birds providing parental care (Stiles et al. 1989; Quesnel 1995; Rochford 2012; Juárez et al. 2024; Cueva and Celorio Mosquera 2025), suggesting a potential female‐limited polymorphism. However, our single observation and lack of morphological examination in hand to confirm the sex and age of the bird limits our ability to draw conclusions about whether the plumage represents a polymorphism or an anomaly, or to discuss underlying mechanisms. Acknowledging these limitations, it is important to note that previous studies have reported: (i) the retention of female tail color patterns in male‐like adult female hummingbirds (Campbell et al. 2024; Falk et al. 2021), and (ii) that parental care behavior is typically confined to female hummingbirds. Based on this context, we explore potential proximate and ultimate mechanisms that could give rise to a female‐limited polymorphism in the Veraguan mango.

Mechanistically, the maintenance of female‐limited polymorphisms in a population requires frequency‐dependent or neutral balancing selection in ecological and social contexts (Falk, Bergstrom, et al. 2025). Here, we speculated on frequency‐dependent balancing selection mechanisms, given the already robust evidence for social drivers of female‐limited polymorphisms in the white‐necked jacobin (Falk et al. 2021; Falk, Bergstrom, et al. 2025). We briefly reflect on three hypothetical mechanisms involving harassment avoidance in younger hummingbirds, interspecific competition for feeding and nesting resources, and variable predation rates of females with male plumage and their nests across different nesting habitats.

### 4.1 Age‐Related Plumage Ontogeny

Falk et al. (2021, 2022) demonstrated that white‐necked jacobin juveniles all appear male‐like. Then, through a post‐juvenile molt, most females attain their typical adult plumage while a minority retain the male‐like plumage. The retention of the juvenile male‐like plumage in a minority of adult females is driven by a social selection mechanism of harassment avoidance by other hummingbirds. In our case, the hummingbird may employ a similar adaptive strategy to reduce harassment from other hummingbirds during a key developmental period, one where investment in body condition and survival at an early age is prioritized over mating. Over time, most females would attain the typical plumage of adult females, and a small percentage would retain the male‐like plumage as adults (Falk et al. 2021). Still unclear are the drivers of the frequency of the polymorphism and whether there are ecological mechanisms also at play based in the natural history of the Veraguan mango.

### 4.2 Social and Ecological Mechanisms

The breeding natural history of species in *Anthracothorax* includes conspicuous nesting in tall trees at forest edges or in open grassy habitats such as savannahs and cattle pastures (Stiles et al. 1989). Small portions of *Anthracothorax* populations utilize urbanized areas for nesting, such as power lines in towns (Juárez et al. 2024). Such urban areas could present favorable conditions for raising young (Reynolds et al. 2019), especially if interspecific competition for foraging and nesting resources is reduced when hummingbirds with similar foraging and nesting ecology are filtered out of urban habitats (Maruyama et al. 2019; Levey et al. 2021, 2025). Before widespread land‐use change throughout *Anthracothorax* distributions, females with relatively conspicuous male plumage may have drawn more attention to predators while foraging and nesting, especially in natural and rural contexts where native predator species are more abundant (Stiles 1978; Kimball and Ligon 1999; Levey et al. 2021). More predators in these contexts may have exerted higher nest predation pressure than current predation rates in highly urban areas (Levey and MacGregor‐Fors 2024). Female mangoes with male plumage that nest in urban areas may benefit from reduced hummingbird harassment and reduced exposure to predation compared to birds that nest in more natural contexts. In this scenario, the frequency of the female‐limited polymorphism would be limited by concentrated flowering resources and by any negative impacts of nesting in highly urbanized areas, such as predation by domestic cats, window collisions, reduced availability of natural insect prey and nectar sources, noise, pollution, and human manipulation of nests (Reynolds et al. 2019; Levey and MacGregor‐Fors 2024).

Despite the uncertainty of the underlying mechanisms, our observation contributes valuable natural history information for the Veraguan mango and opens the possibility of a female‐limited polymorphism in this hummingbird species. Our discussion of potential mechanisms could open further study into the Veraguan and other mango species to determine the frequency of female‐limited polymorphisms in populations and the ecological drivers of female‐limited polymorphisms, including the role of variable predation rates of male‐plumaged adult female hummingbirds and their nests along land‐use gradients.

## Author Contributions

**Dallas R. Levey:** conceptualization (lead), formal analysis (lead), investigation (lead), methodology (lead), visualization (lead), writing – original draft (lead). **Gretchen C. Daily:** conceptualization (supporting), funding acquisition (lead), methodology (supporting), visualization (supporting), writing – original draft (supporting).

## Conflicts of Interest

The authors declare no conflicts of interest.

## Data Availability

Full video referenced in the article is available on Dryad (https://doi.org/10.5061/dryad.jh9w0vtp7; Levey and Daily 2025).
